# Contraception Use in Patients With Unfulfilled Permanent Postpartum Contraception Requests

**DOI:** 10.7759/cureus.84047

**Published:** 2025-05-13

**Authors:** Cheryl Godcharles, Megan Carney, Amanda Lacue, James D Hickman, Lisa Perriera, Rebecca Mercier

**Affiliations:** 1 Department of Obstetrics and Gynecology, University of South Florida, Tampa, USA; 2 Department of Neonatology, Mount Sinai Medical Center, New York City, USA; 3 Department of Obstetrics and Gynecology, University of Pittsburgh Medical Center, Pittsburgh, USA; 4 Department of Obstetrics and Gynecology, Thomas Jefferson University, Philadelphia, USA

**Keywords:** bilateral tubal ligation, birth control, family planning, salpingectomy, sterilization

## Abstract

Introduction

Permanent contraception with tubal surgery is commonly used and a highly effective form of contraception. The postpartum period is a safe, effective, and convenient time for permanent contraception. Our study looked to investigate postpartum permanent contraception (PPC) use and how unfulfilled requests affect overall contraceptive use.

Objective

The primary aim of this study is to determine whether patients who have unfulfilled PPC requests at our institution are as likely as patients who do not desire PPC to use an effective method of contraception in the postpartum period.

Study design

Data was abstracted from the charts of all patients delivering viable infants at a large, urban, academic hospital throughout one year. The primary outcome was contraceptive use at the postpartum visit for those patients who did not want or did not get PPC. Secondary outcomes included: contraceptive use at hospital discharge, contraceptive use one year after delivery, and repeat pregnancy rates.

Results

A total of 1,894 women delivered viable infants during the study period; 1,671 (88.23%) did not request permanent contraception. Of the remaining 223 women, 132 (6.97%) received PPC during their delivery admission, and 91 (4.8%) did not. Of women who did not receive PPC, 48.35% were using some form of contraception at discharge and 81.43% were using contraception at the end of the study period; this is compared to 73.93% and 90.16% in the control group at each time point, respectively.

Conclusion

Women with unfulfilled requests for PPC are significantly less likely to use any form of contraception at hospital discharge, postpartum visit, and one year from delivery compared to women who did not desire PPC.

## Introduction

Nearly 10 million individuals in the United States use tubal surgery for contraception, making it one of the most commonly used forms of contraception [1]. The postpartum period is a safe, effective, and convenient time for tubal surgery, and half occur during the delivery admission [2-3]. Delivering patients often prefer to obtain contraception during their delivery hospitalization rather than at a return visit. Despite this, 30-50% of patients who express a desire for postpartum permanent contraception (PPC) do not undergo the procedure prior to hospital discharge, leaving over 350,000 patients every year without timely access to their desired form of contraception [4-9].

Barriers to obtaining PPC include lack of a valid Medicaid sterilization consent, medical reasons (including anemia and obesity), unavailable operating rooms, and patient requests to defer [6,9-11]. One study focusing on low-income, minority women also identified sense of autonomy as well as relationship with provider and hospital staff during delivery as reasons why patients deferred a PPC procedure [10]. Multiple studies find the absence of a valid, signed Medicaid consent form is the most common reason for unfulfilled postpartum tubal requests [6,9]. One study in Chicago, Illinois, found that of the 231 patients who had a documented plan for postpartum permanent contraception, 51.1% underwent the procedure immediately postpartum, 6.9% underwent the procedure in the year follow-up period, and 42.0% did not have the procedure performed at all. Additionally, patients with high-risk pregnancies were more likely to desire postpartum tubal surgery as their contraceptive method, although these patients were no more likely to undergo the procedure immediately postpartum or at any time during the follow-up period [12].

Other studies looked at follow-up rates of completed permanent contraception procedures and repeat pregnancies. Flink-Bochacki et al. explored contraceptive and reproductive outcomes of patients who experienced unfulfilled PPC requests. In this cohort, fewer than half of the patients requesting PPC successfully underwent the requested procedure by the one-year follow-up, and 22.8% of those became pregnant during the next year. This study found that institutional policies created many obstacles to postpartum permanent contraception [13]. Similarly, Thurman and Janecek evaluated one-year follow-up of patients requesting PPC and compared repeat pregnancy rates and patterns of contraceptive use amongst those with unfulfilled PPC requests and those who did not desire PPC. The study population was primarily undocumented United States residents, which likely affected these patients’ access to the healthcare system and their contraceptive practices. In this population, individuals with unfulfilled PPC were significantly more likely to have a pregnancy within one year. They also requested specific contraceptive methods at similar rates to those who did not desire PPC [4].

The patients at our large urban hospital represent a diverse group of people, most of whom have access to the healthcare system for at least six weeks after delivery. Unfulfilled PPC requests seem to have a big impact on our patients. The goal of this study is to evaluate the differences in contraceptive use and rapid repeat pregnancy rates between patients with unfulfilled PPC requests and those not requesting PPC. Unfulfilled PPC requests refer to patients who requested but did not receive postpartum contraception.

## Materials and methods

The study was constructed as a comparative, retrospective cohort study at Thomas Jefferson University Hospital, a large university teaching medical center in Philadelphia, Pennsylvania. The institution is a tertiary care center with approximately 2000 annual deliveries and serves a diverse patient population, including a high proportion of publicly insured and medically underserved individuals. Institutional Review Board approval was obtained prior to the initiation of this study.

Study charts were identified through a report of all deliveries between October 1, 2016, and September 30, 2017, using the electronic medical record. The end of the study period for each patient was defined as one year from the date of delivery to allow for assessment of longitudinal outcomes such as contraceptive use and repeat pregnancy. Patients were included if they had a viable pregnancy and underwent delivery at our institution during the specified time frame. Patients with nonviable pregnancies (e.g., intrauterine fetal demise, miscarriage), multiple deliveries within the study period, or deliveries occurring at outside institutions were excluded from the study. 

Data obtained at the time of chart review included age, gravidity/parity, ethnicity, race, type of insurance, body mass index at delivery, mode of delivery, gestational age at delivery, day of the week of delivery, and PPC request. We also noted the number of follow-up visits, if there was another pregnancy within the study period, and all contraception used throughout the study period, including at hospital discharge and postpartum visit. If a patient requested PPC, we documented the timing of the request (antepartum, delivery admission, postpartum) and when the patient underwent the procedure. If PPC was completed, we recorded the specific surgical technique used (e.g., partial salpingectomy, total salpingectomy, tubal ligation).

The primary outcome was contraceptive use at the postpartum visit for those patients who did not want or did not get PPC. We defined contraceptive use as any of the following: completed long-acting reversible contraception (LARC) placement, contraceptive injection given, contraceptive prescription provided, and/or patient-reported intent to use non-prescription methods. Secondary outcomes included: contraceptive use at hospital discharge, contraceptive use one year after delivery, and repeat pregnancy rates.

We used descriptive statistics to characterize the study and control groups. Bivariable analyses were used to compare baseline characteristics and outcomes between groups. The primary and secondary outcomes were also compared using the Pearson chi-square test. To adjust for potential confounding variables (e.g., age, insurance type, parity), we performed multivariable logistic regression to estimate the odds of contraceptive use at the postpartum visit. Results were reported as adjusted odds ratios (aORs) with 95% confidence intervals (CIs).

All analyses were performed using STATA 20, with statistical significance defined as p < 0.05. 

## Results

A total of 1,894 patients delivered viable infants during the study period. A total of 223 (11.8%) requested PPC; of these, 132 (59%) received PPC while 91 (49%) did not. Patients with unfulfilled PPC requests were more likely to be older, have Medicaid as their primary insurance, have a higher BMI, have a higher gravidity and parity, be single, deliver via cesarean, deliver preterm, be Hispanic, and be on opioid maintenance therapy. These patients were less likely to be Asian/Pacific Islander (Table 1). The mean gestational age at delivery for patients requesting and not requesting PPC was not statistically different.

**Table 1:** Population characteristics (mean [SD] or N [%]) PPC: Permanent postpartum contraception * Statistically significant p-value

	Fulfilled PPC N=132 Mean (SD)	Unfulfilled PPC N=91 Mean (SD)	No PPC Request N=1671 Mean (SD)	Test statistic F value	P-value
Age (years)	33.3 (4.4)	32.3 (4.5)	28.99 (5.6)	70.92	<0.001*
Gravidity (mean total pregnancy)	4.7 (2.5)	5.0 (2.4)	2.3 (1.9)	150.32	<0.001*
Parity (mean prior live birth)	3.1 (1.4)	3.5 (1.5)	1.8 (1.2)	185.21	<0.001*
Body Mass Index	33.5 (7.8)	33.7 (7.3)	31.5 (6.5)	11.54	<0.001*
Weeks gestation at delivery	37.4 (2.7)	37.9 (2.1)	38.5 (9.0)	1.79a	0.167
	Fulfilled PPC N=132 N (%)	Unfulfilled PPC N=91 N (%)	No PPC Request N=1671 N (%)	Test statistic X^2^	P value
Race				24.53	<0.001*
White	53 (40.1)	27 (29.7)	638 (38.2)		
Black	61 (46.2)	53 (58.2)	672 (40.2)		
Asian	11 (8.3)	7 (7.7)	312 (18.7)		
Other	5 (3.8)	3 (3.3)	33 (2.0)		
Hispanic Ethnicity	22 (16.6)	11 (12.1)	119 (7.1)	17.24	<0.001*
Insurance				22.42	
Medicaid	83 (62.8)	70 (76.9)	923 (55.4)		0.002*
Commercial	42 (31.8)	20 (22.0)	691 (41.5)		
Other	2 (1.5)	0 (0)	34 (2.0)		
None	3 (2.3)	1 (1.1)	16 (1.0)		
Married	40 (30.3)	22 (24.2)	627 (37.5)	8.9	0.013*
Planned Pregnancy	25 (18.9)	10 (11.0)	399 (23.9)	9.39	<0.001*
Opiate maintenance therapy	19 (14.3)	9 (9.9)	126 (7.6)	8.09	0.018*
Preterm delivery	35 (26.5)	13 (14.2)	236 (14.1)	14.78	<0.001*
Mode of delivery				130.73	
Vaginal	35 (26.5)	80 (87.9)	1186 (71.3)		<0.001*
Cesarean	97 (73.5)	11 (12.1)	477 (28.6)		

Of patients who did not receive PPC, 48.4% obtained some form of contraception at discharge compared to 73.9% of patients who did not request PPC (p<0.001) (Table 2). A total of 67.1% of patients with unfulfilled PPC presented for a postpartum visit compared to 85.3% of patients who did not request PPC (p<0.001). At the end of their postpartum visit, 74.4% of patients who had an unfulfilled PPC request and 91.4% of patients who did not request PPC were using contraception (p<0.001). Additionally, 81.4% of patients with unfulfilled PPC requests were using contraception at the end of the study period compared to 90.1% of patients without PPC requests (p=0.019). Notably, 19 patients who had requested a tubal and did not receive PPC during their hospital admission had tubal surgery completed by the end of the study period (27.1%).

**Table 2:** Contraceptive use and rapid repeat pregnancy (N [%]) PPC: Permanent postpartum contraception * Statistically significant p-value

	Unfulfilled PPC Request N (%)	No PPC Request N (%)	P-value	Odds Ratio (95% CI)
On hospital discharge			<0.001	0.301 (0.158-0.573)
Yes	44 (48.4)	1231 (73.6)		
No	47 (51.7)	434 (25.9)		
At postpartum visit			<0.001	0.188, (0.74-0.476)
Yes	35 (74.4)	1021(91.4)		
No	12 (32.4)	96 (8.6)		
End of study period			0.019	0.333, (0.150-0.741)
Yes	57 (81.4)	1182 (90.2)		
No	13 (18.5)	129 (9.8)		
Rapid repeat pregnancy	5 (7.1)	82 (6.2)	0.759	1.16 (0.45-2.95)

We observed no significant interaction or confounding effects. In logistic regression analysis, patients who did not receive requested PPC were significantly less likely to be using contraception compared to patients who did not request PPC at hospital discharge (OR: 0.301, 95% CI: 0.158-0.573), postpartum visit (OR: 0.188, 95% CI: 0.74-0.476), and the end of the study period (OR: 0.333, 95% CI: 0.150-0.741).

Five patients with unfulfilled requests for permanent contraception had a rapid repeat pregnancy within the study period (7.14%) compared to 82 patients in the control group (6.23%).

## Discussion

Principal findings

We found that patients who had unfulfilled requests for PPC at discharge from delivery were less likely than patients who did not request PPC to use contraception at hospital discharge, postpartum visit, and at the end of a one-year study period. There was no significant difference in rapid repeat pregnancy rates between the two groups. 

Results

Forty percent of patients in our population who requested PPC received it prior to discharge from their delivery admission, which is similar to the 44% rate published by Seibel-Seamon in 2009. Seibel-Seamon found that 44% of patients with unfulfilled PPC requests had an interval tubal surgery [14]. In our population, much fewer interval surgeries were completed. 

The American College of Obstetricians and Gynecologists (ACOG) recognizes completion of postpartum tubal ligations as an important strategy for reducing unintended pregnancy rates. ACOG recommends that these surgeries be classified as urgent rather than elective, given the short time frame in which they may be performed and the individual and public health consequences associated with unfulfilled PPC requests [2].

Clinical implications

This study further demonstrates the need to prioritize the completion of postpartum tubal surgery. Many patients who express a desire for permanent contraception are not using any form of contraception after being denied the option that they originally desired. Patients with unfulfilled requests for permanent contraception are more likely to decline an interval or substitute contraceptive method. It is necessary for providers to continue working to overcome barriers to PPC completion in order to provide patients with the effective contraceptive of their choice. Increasing access to interval tubal surgery may also help further increase the percentage of individuals using contraception. The ExITS study recently demonstrated that patients who undergo expedited scheduling of interval tubal are 10 times more likely to have their surgery completed than patients who must wait for the procedure [15].

In our study, patients with unfulfilled PPC requests were more likely to have a higher BMI compared to those who were able to undergo surgery for PPC. While the rate of general surgical complications tends to be higher in obese patients, one study found no association between increased BMI and morbidity when focusing on postpartum tubal ligation procedures specifically. The difference in composite morbidity in their study of over 3,000 patients was not statistically significant across women with BMI less than 25 up through women with BMI greater than 40 [11]. BMI alone should not be a reason to withhold PPC from patients. 

It is also important to readdress contraceptive education for patients with unfulfilled PPC requests. This is a population of patients that has expressed the desire to permanently prevent pregnancy, and there should be emphasis on delivering highly effective methods of contraception to them. Many patients who do not receive their desired tubal PPC may be eligible for postpartum placement of a contraceptive implant or intrauterine device prior to hospital discharge. Every patient should receive comprehensive contraceptive counseling during the prenatal period; those requesting PPC should be counseled about the rate of unfulfilled PPC requests at the institution and be asked to create a backup plan if they cannot get the PPC. Care should be taken to avoid coercion and biases within this counseling. 

Research implications

Further research is needed to explore reasons why patients with unfulfilled requests for PPC do not choose alternative contraceptive methods as often as patients do not desire PPC. A larger study evaluating the difference in LARC use between patients requesting PPC and those who do not could help obstetricians better meet the postpartum family planning needs of their patients. 

Strengths and limitations 

Strengths of this study include the large sample size and a racially and ethnically diverse population. Limitations of this study include a short study period; perhaps a longer study period would have been more effective in evaluating repeat pregnancies and their outcomes (including whether or not they were planned and desired). We collected data from a single hospital in an urban setting. Additionally, our institution is the delivery site for a local health center, so while we have data from delivery admission, we do not have access to postpartum follow-up data (including contraceptive use) for those patients.

## Conclusions

In summary, many patients do not receive the requested permanent contraception during their delivery admission. These patients are significantly less likely to be using contraception at hospital discharge, at the time of postpartum visit, and one year postpartum. This highlights an essential area for improvement in our provision of obstetric and gynecologic care. Specifically, an intentional and targeted focus on expediting postpartum tubal ligation procedures during delivery admissions is needed. Further studies are needed to investigate reasons why postpartum tubal ligations were not completed and interventions to improve access to this care.
